# Supplementary material for: Low-Temperature Processed TiOx/Zn1−xCdxS Nanocomposite for Efficient MAPbIxCl1−x Perovskite and PCDTBT:PC70BM Polymer Solar Cells
Source: Polymers (Basel). 2019 Jun 3;11(6):980. doi: 10.3390/polym11060980 (PMC6631563; doi:10.3390/polym11060980)
Supplement: Supplementary file 1 [file polymers-11-00980-s001.pdf]

Supplementary Information

Low-Temperature Processed $TiO_x/Zn_{1-x}Cd_xS$ Nanocomposite for Efficient $MAPbI_xCl_{1-x}$ Perovskite and $PCDTBT:PC_{70}BM$ Polymer Solar Cells

Binh Duong,[1] Kathawut Lohawet, [1] Tanyakorn Muangnapoh, [1] Hideki Nakajima, [2] Narong Chanlek, [2] Anirudh Sharma, [3] David Lewis,[4] Pisist Kumnorkaew[1,]*

[1] National Nanotechnology Center, National Science and Technology Development Agency, Pathum Thani, 12120, Thailand

[2] Synchrotron Light Research Institute, Nakhon Ratchasima, 30000, Thailand

[3] University of Bordeaux, Laboratoire de Chimie des Polymères Organiques (LCPO), UMR 5629, B8 Allée Geoffroy Saint Hilaire, 33615 Pessac Cedex, France.

[4] Flinders Centre for Nanoscale Science and Technology, Flinders University, Adelaide, SA 5042, Australia

Results

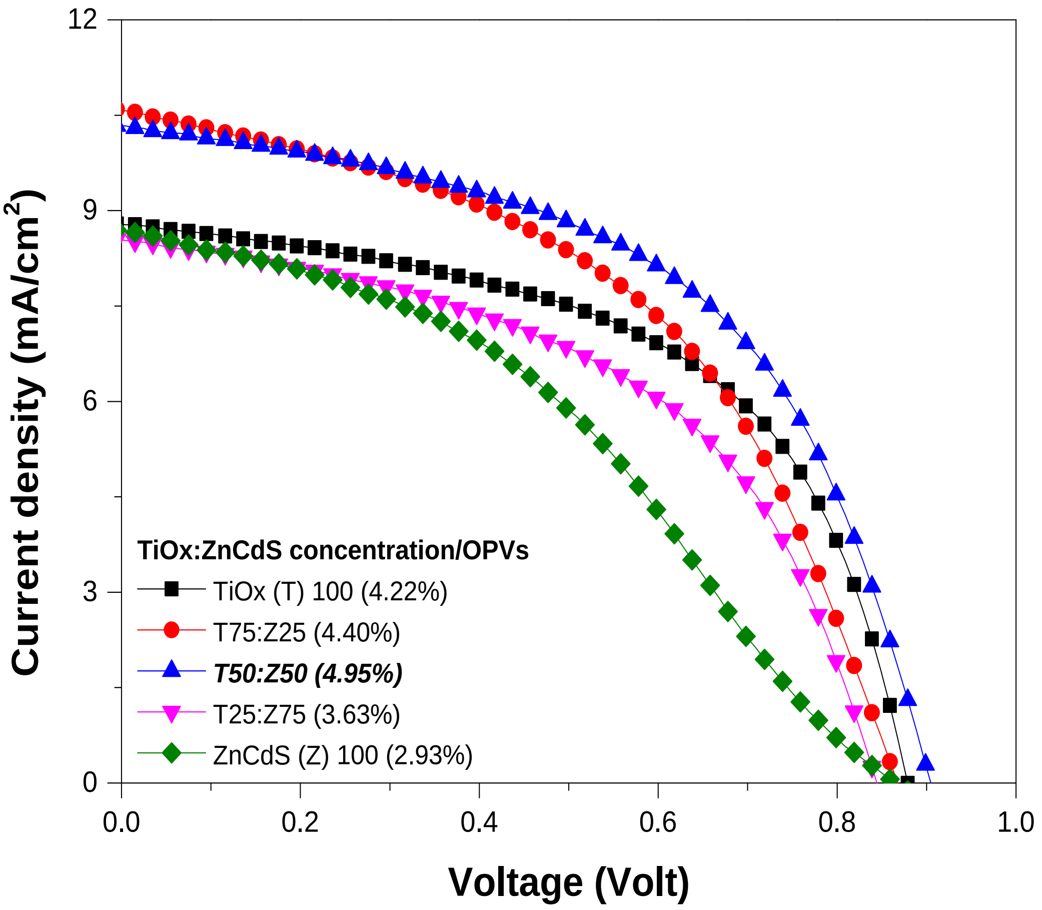

Figure S1. J-V characteristics of polymer solar cells

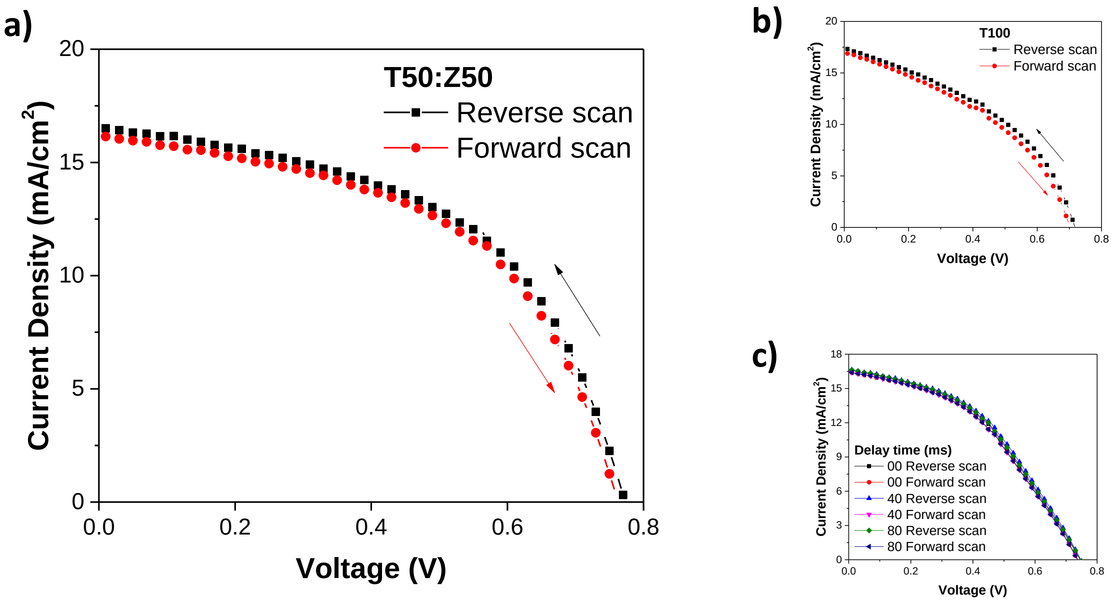

Figure S2. Hysteresis study of p-i-n perovskite solar cells device (**a**) the forward-reverse J-V curves with 40 ms delay time of T50:Z50 ETL. (**b**) the forward-reverse J-V curves with 40 ms delay time of 100 ETL. (**c**) the forward-reverse J-V curves with 0, 40 and 80 ms delay time of T100 ETL with negligible hysteresis .

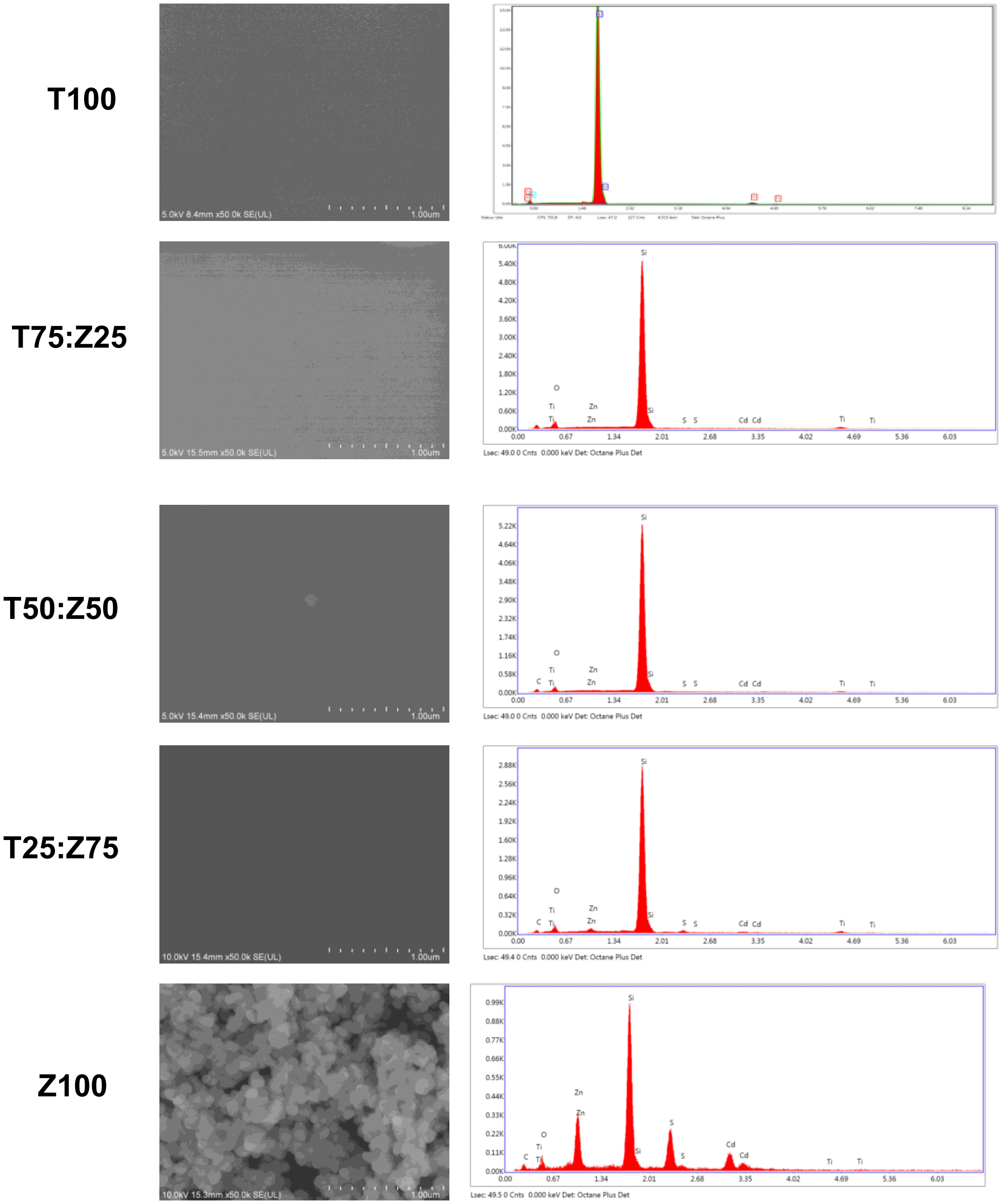

Figure S3. SEM and corresponding EDS spectra of T:Z nanocomposite films.

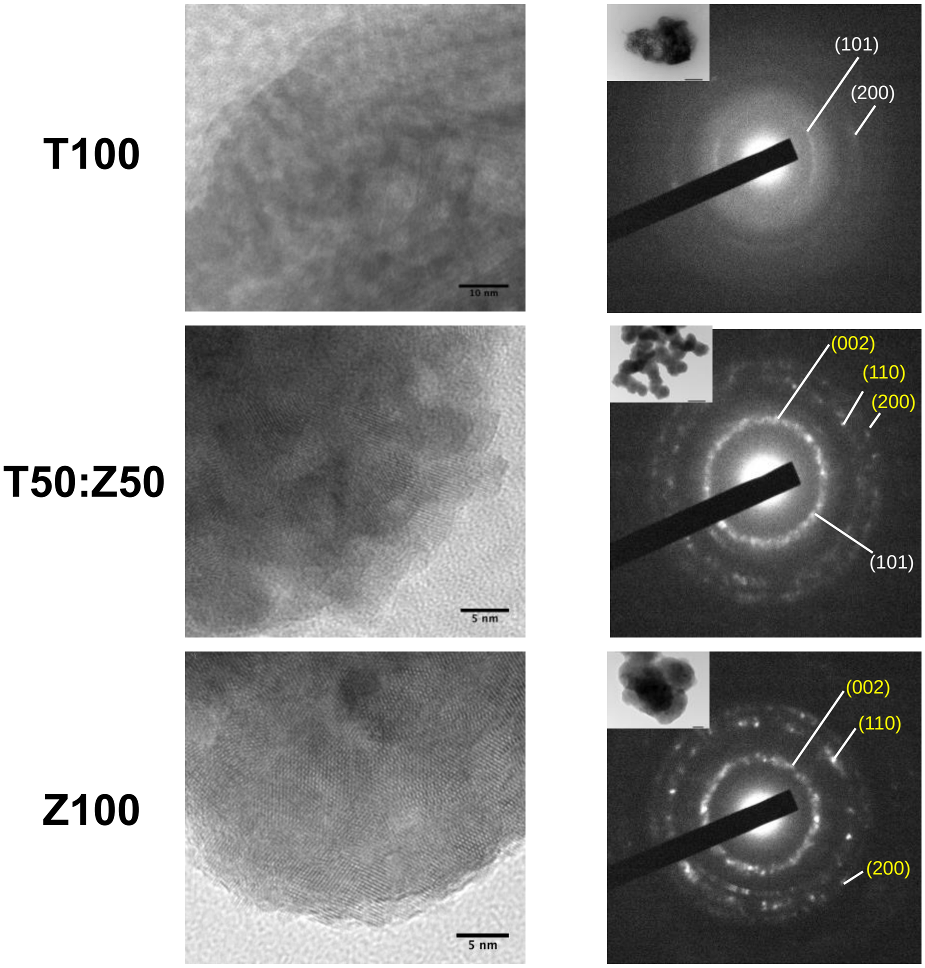

Figure S4. High-resolution TEM images and diffraction patterns at selected areas (insets) from of T100, T50:Z50 and Z100 samples.

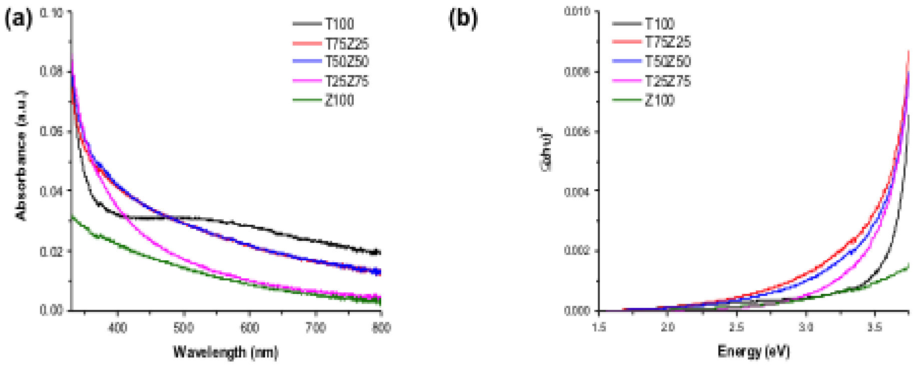

Figure S5. (**a**) UV-Vis spectra and (**b**) Tau plot of ETL composite thin films.

Table S1. Surface potential difference and work function of T:Z composite films obtained from SKPM technique.

	**Vcpd (mV)**	**Work function (eV)**
**T100**	292.03 ± 0.44	3.47 ± 0.44
**T75Z25**	356.28 ± 0.48	4.21 ± 0.48
**T50Z50**	258.23 ± 0.28	3.75 ± 0.28
**T25Z75**	358.80 ± 0.36	4.67 ± 0.36
**Z100**	138.92 ± 0.29	3.77 ± 0.29

Table S2. Binding energy and work function of T:Z composite films obtained from UPS technique. Photon energy is 40.8 eV.

	**Binding energy cutoff (eV)**	**Work function (eV)**
**T100**	36.58	4.22
**T75Z25**	36.80	4.00
**T50Z50**	37.10	3.70
**T25Z75**	36.72	4.08
**Z100**	37.20	3.60

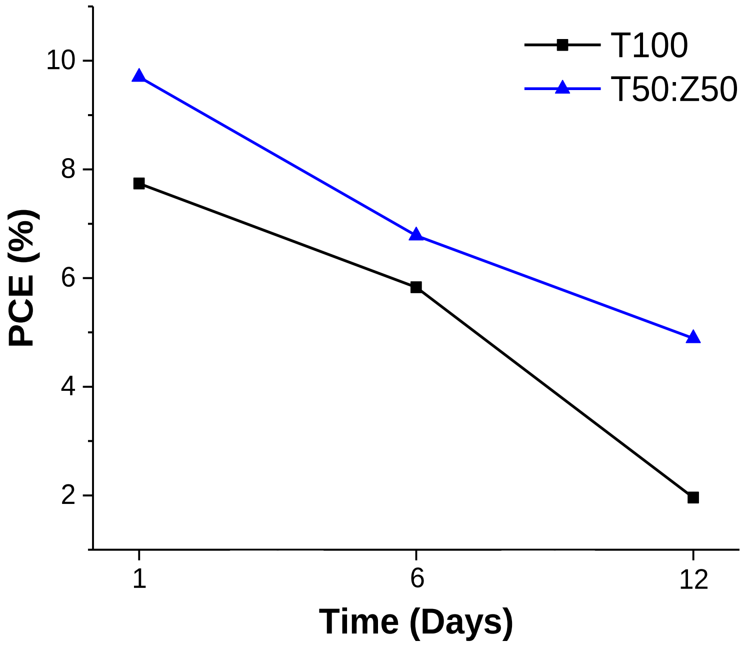

Figure S6 Stability of perovskite solar cell over 12 days**.**

Table S3. Perovskite solar cell parameters of with T100 and T50:Z50 ETL over 12 days.

Time (Days)	V_{oc} (v)	J_{sc} (mA/cm²)	*FF*	*PCE* (%)	Area (cm²)
T100 (D1)	0.859	15.14	0.60	7.74	0.1
T100 (D6)	0.901	11.95	0.54	5.83	0.1
T100 (D12)	0.767	5.96	0.43	1.96	0.1
T50:Z50 (D1)	0.889	17.99	0.61	9.79	0.1
T50:Z50 (D6)	0.874	13.98	0.55	6.78	0.1
T50:Z50 (D12)	0.823	13.19	0.45	4.89	0.1
